# Comparative Significance of Invasive Measures of Microvascular Injury in Acute Myocardial Infarction

**DOI:** 10.1161/CIRCINTERVENTIONS.119.008505

**Published:** 2020-05-15

**Authors:** Annette M. Maznyczka, Keith G. Oldroyd, John P. Greenwood, Peter J. McCartney, James Cotton, Mitchell Lindsay, Margaret McEntegart, J. Paul Rocchiccioli, Richard Good, Keith Robertson, Hany Eteiba, Stuart Watkins, Aadil Shaukat, Colin J. Petrie, Aengus Murphy, Mark C. Petrie, Colin Berry

**Affiliations:** 1 British Heart Foundation Glasgow Cardiovascular Research Centre, Institute of Cardiovascular and Medical Sciences, University of Glasgow, United Kingdom (A.M.M., K.G.O., P.J.M., M.C.P., C.B.).; 2 West of Scotland Heart and Lung Centre, Golden Jubilee National Hospital, Clydebank, Glasgow, United Kingdom (A.M.M., K.G.O., P.J.M., M.L., M.McE., J.P.R., R.G., K.R., H.E., S.W., A.S., C.B.).; 3 Leeds University and Leeds Teaching Hospitals NHS Trust, United Kingdom (J.P.G.).; 4 Wolverhampton University Hospital NHS Trust, United Kingdom (J.C.).; 5 University Hospital Monklands, NHS Lanarkshire, United Kingdom (C.J.P.).

**Keywords:** heart failure, hospitalization, magnetic resonance imaging, microcirculation, myocardial infarction

## Abstract

The resistive reserve ratio (RRR) expresses the ratio between basal and hyperemic microvascular resistance. RRR measures the vasodilatory capacity of the microcirculation. We compared RRR, index of microcirculatory resistance (IMR), and coronary flow reserve (CFR) for predicting microvascular obstruction (MVO), myocardial hemorrhage, infarct size, and clinical outcomes, after ST-segment–elevation myocardial infarction.

**Methods:**

In the T-TIME trial (Trial of Low-Dose Adjunctive Alteplase During Primary PCI), 440 patients with acute ST-segment–elevation myocardial infarction from 11 UK hospitals were prospectively enrolled. In a subset of 144 patients, IMR, CFR, and RRR were measured post-primary percutaneous coronary intervention. MVO extent (% left ventricular mass) was determined by cardiovascular magnetic resonance imaging at 2 to 7 days. Infarct size was determined at 3 months. One-year major adverse cardiac events, heart failure hospitalizations, and all-cause death/heart failure hospitalizations were assessed.

**Results:**

In these 144 patients (mean age, 59±11 years, 80% male), median IMR was 29.5 (interquartile range: 17.0–55.0), CFR was 1.4 (1.1–2.0), and RRR was 1.7 (1.3–2.3). MVO occurred in 41% of patients. IMR>40 was multivariably associated with more MVO (coefficient, 0.53 [95% CI, 0.05–1.02]; *P*=0.031), myocardial hemorrhage presence (odds ratio [OR], 3.20 [95% CI, 1.25–8.24]; *P*=0.016), and infarct size (coefficient, 5.05 [95% CI, 0.84–9.26]; *P*=0.019), independently of CFR≤2.0, RRR≤1.7, myocardial perfusion grade≤1, and Thrombolysis in Myocardial Infarction frame count. RRR was multivariably associated with MVO extent (coefficient, −0.60 [95% CI, −0.97 to −0.23]; *P*=0.002), myocardial hemorrhage presence (OR, 0.34 [95% CI, 0.15–0.75]; *P*=0.008), and infarct size (coefficient, −3.41 [95% CI, −6.76 to −0.06]; *P*=0.046). IMR>40 was associated with heart failure hospitalization (OR, 5.34 [95% CI, 1.80–15.81] *P*=0.002), major adverse cardiac events (OR, 4.46 [95% CI, 1.70–11.70] *P*=0.002), and all-cause death/heart failure hospitalization (OR, 4.08 [95% CI, 1.55–10.79] *P*=0.005). RRR was associated with heart failure hospitalization (OR, 0.44 [95% CI, 0.19–0.99] *P*=0.047). CFR was not associated with infarct characteristics or clinical outcomes.

**Conclusions:**

In acute ST-segment–elevationl infarction, IMR and RRR, but not CFR, were associated with MVO, myocardial hemorrhage, infarct size, and clinical outcomes.

**Registration:**

URL: https://www.clinicaltrials.gov; Unique identifier: NCT02257294.

## Abstract 2

Supplemental Digital Content is available in the text.

**What Is Known**

- Invasive physiology parameters that predict microvascular obstruction and prognosis may become useful for selection of patients for adjunctive therapies during primary percutaneous coronary intervention.
- Resistive reserve ratio is a measure of the vasodilator capacity of the microcirculation, whereas index of microcirculatory resistance is a measure of the minimum achievable microvascular resistance at peak hyperemia.
- There is a lack of data on the comparative significance of established and novel invasive measures of coronary vascular function, for predicting microvascular injury and prognosis, in patients with acute ST-segment–elevation myocardial infarction.

**What the Study Adds**

- Index of microcirculatory resistance and resistive reserve ratio reflect different aspects of microvascular function, are complementary and are associated with microvascular obstruction extent, myocardial hemorrhage presence, infarct size and clinical outcomes.
- Our findings support using the index of microcirculatory resistance in conjunction with resistive reserve ratio instead of coronary flow reserve, as a tool to select patients for adjunctive therapy during primary percutaneous coronary intervention.

**See Editorial by Jeremias and Ali**

Immediate coronary revascularization by primary percutaneous coronary intervention (PCI) is the standard of care for patients with ST-segment–elevation myocardial infarction (STEMI).^1^ Despite routinely restoring epicardial coronary blood flow, about half of patients have impaired myocardial perfusion.^2^ Invasive measures of microvascular dysfunction in the culprit artery have potential to guide the selection of patients for adjunctive therapies during primary PCI.^3^

Microvascular damage can be quantified on cardiovascular magnetic resonance (CMR), where it is referred to as microvascular obstruction (MVO), and is prognostically important.^4^ However, CMR is not feasible immediately post-reperfusion. Invasive coronary physiology parameters provide an immediate assessment of post-PCI microvascular function. Among these parameters, the index of microcirculatory resistance (IMR) has been validated in animals^5,6^ and in humans.^7–10^ Higher IMR values indicate greater degrees of microvascular dysfunction^7,11^ and an IMR>40 predicts worse clinical outcomes (death, hospitalization for heart failure, and MI).^11,12^

Coronary flow reserve (CFR) reflects epicardial and microcirculatory vasodilator capacity, as well as residual epicardial stenosis. In acute STEMI, a lower CFR predicts MVO and larger infarction.^7,13^ However, compared with IMR>40, the combination of IMR>40 and CFR≤2.0 did not have incremental prognostic value.^12^

The resistive reserve ratio (RRR)^14–16^ is a newer, less well-studied parameter. RRR is derived as the ratio between basal resting tone in the microcirculation and microcirculatory resistance at maximal hyperemia.^14^ RRR describes the ability of the coronary microcirculation to vary its resistance in response to a hyperemic stimulus, for example, adenosine.^14^ Higher RRR values indicate greater vasodilatation of the microcirculation in response to hyperemia, while lower RRR values indicate poor vasodilator capacity of the coronary microcirculation. RRR is a measure of the microvascular vasodilatory response, which integrates measurement of pressure (microvascular resistance), thereby RRR is theoretically distinct from CFR. In contrast, IMR does not reflect microvascular vasodilator capacity. Whether these differences may be clinically significant is uncertain.

We predefined this physiology substudy of the T-TIME trial (Trial of Low-Dose Adjunctive Alteplase During Primary PCI). The principle aim was to compare the associations of IMR, CFR, and RRR with MVO extent (a reference surrogate outcome measure of failed microvascular reperfusion). Second, we compared the associations of IMR, CFR, and RRR with myocardial hemorrhage, infarct size, and clinical outcomes. We hypothesized that IMR, CFR, and RRR would be associated with infarct characteristics and clinical outcomes, and that RRR would more closely associate with microvascular dysfunction than CFR.

## Methods

The data that support the findings of this study are available from the corresponding author upon reasonable request.

The patients were enrolled into the prespecified physiology substudy of the T-TIME trial,^17^ which was a double-blind randomized clinical trial of adjunctive intracoronary alteplase (10 or 20 mg) versus placebo delivered post-reperfusion, but pre-stenting, and found no difference in MVO at 2 to 7 days. From 2016 to 2017, 144 patients with STEMI ≤6 hours from symptom onset, from 3 UK hospitals, were enrolled. Eligibility criteria (Data Supplement) included occlusion or reduced flow (Thrombolysis in Myocardial Infarction [TIMI] coronary flow grade ≤2) in the culprit artery, with thrombus evident angiographically. The study was approved by the National Research Ethics Service (13-WS-0119) and complied with the Declaration of Helsinki. Witnessed verbal assent to participate was obtained in the catheterization laboratory. Written informed consent was subsequently obtained on the ward.

### Invasive Coronary Physiology

IMR, CFR, and RRR were measured at the end of primary PCI using a pressure- and temperature-sensing guidewire (Abbott, Vascular, CA). Intracoronary nitroglycerin (200 µg) was administered into the culprit artery. A calibrated wire was equalized to guide catheter pressure, then advanced to the distal third of the culprit artery. Using standard thermodilution methods, the mean transit time (Tmn) of a hand-injected 3 mL bolus of room temperature saline was measured in triplicate at rest and during steady-state maximal hyperemia, induced by intravenous adenosine (140 µg/kg per minute). Simultaneous measurements of Pa and Pd were made.

IMR was defined as Pd×Tmn during hyperemia.^5^ When IMR was measured after stenting, there was no residual epicardial stenosis, and therefore IMR correction with wedge pressure,^18^ or Yong’s formula^19^ was not required. A threshold of 40 was used to dichotomize IMR in regression analyses because based on published literature an IMR>40 is prognostically significant. CFR was quantified by dividing resting Tmn by hyperemic Tmn.^20^ A threshold of 2.0 was used to dichotomize CFR in regression analyses because based on published literature a CFR≤2.0 is considered abnormal.^12^

Baseline resistance index (BRI) is a measure of the resting tone in the coronary microcirculation and was calculated using the following previously validated equation^14^:


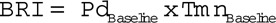


To measure the ability of the coronary microcirculation to undergo vasodilatation in response to a pharmacological hyperemic stimulus, the RRR was calculated as previously described:


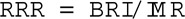


RRR measures the ability of the coronary microcirculation to change from baseline to minimal resistance in response to adenosine, thereby RRR reflects the ability to achieve maximal hyperemia. There are no established cutoffs for RRR; therefore, RRR was dichotomized by the median value, which is the conventional approach taken by previous studies.^15^

To mitigate potential bias through disclosure of coronary physiology results, operators were blinded, by obscuring the display of the RadiAnalyzer Xpress monitor. Experienced physiology technicians recorded the thermodilution data and quality assured the acquisition. Data were extracted from the RadiAnalyzer Xpress instrument and analyzed offline (Coroventis Research AB, Uppsala, Sweden).

The coronary physiology parameters were calculated prospectively and were submitted to the data coordination center before data lock. The coronary physiology analyses were performed by an observer blinded to the CMR data, and vice versa.

### Angiogram Analyses

Angiographic end points were determined by blinded core laboratory analysis. Angiographic analyses included the following: TIMI coronary flow grade, corrected TIMI frame count (TFC), myocardial perfusion grade (MPG), and TIMI thrombus grade in the culprit artery (Methods in the Data Supplement).

### Cardiovascular Magnetic Resonance

CMR imaging was performed at 1.5-Tesla. MVO was reported at 2 to 7 days. CMR was also performed at 3 months. The CMR protocol has previously been described in detail.^7,12,17^ MVO presence and extent, and infarct size, (% left ventricle mass) were demonstrated by late gadolinium enhancement images. Myocardial hemorrhage presence and extent (% left ventricle mass) was demonstrated by T2* mapping.

### Clinical Outcomes

Information on serious adverse events during follow-up was obtained by site research staff. These events were reviewed and adjudicated by the clinical events committee, comprising of 3 cardiologists who were independent of the trial. Clinical events were assessed at 1 year.

We prespecified clinical outcomes that are pathophysiologically linked with the natural history of STEMI. The clinical outcomes were the following: (1) heart failure hospitalization; (2) all-cause death and heart failure hospitalization; and (3) major adverse cardiac events (MACE), defined as cardiac death, nonfatal MI, or hospitalization for heart failure. Clinical follow-up was completed for all subjects.

Hospitalization for heart failure was defined as the following: (1) new or worsening signs/symptoms of heart failure requiring the initiation of, or increase in heart failure directed treatment (including intravenous therapy), or occurring in a patient already receiving maximal heart failure therapy, or (2) confinement to bed predominantly due to heart failure symptoms, or (3) pulmonary edema sufficient to cause tachypnoea and distress (not occurring in the context of an acute MI, worsening renal function [that is not wholly explained by worsening heart failure], or as the consequence of arrhythmia without worsening heart failure), or (4) cardiogenic shock.

### Statistics

Continuous data were summarized using mean±SD, or median and interquartile ranges if skewed. Categorical variables were reported as frequency and percentages. The associations between coronary physiology parameters and MVO extent were assessed by linear regression and were adjusted for the following covariates: TFC post-PCI, MPG≤1 post-PCI and other coronary physiology parameters, that is, CFR dichotomized by 2, RRR dichotomized by median, and IMR dichotomized by 40. There was a priori concern that these covariates were clinically relevant confounders. Continuous coronary physiology parameters were not included as covariates together in the same model, due to collinearity. The regression coefficients from linear regression represented mean change in the extent of the outcome for a 1-unit increase in the predictor. The validity of linear and logistic regressions was verified by analysis of model residuals, linearity condition, testing for heteroscedasticity, and multicollinearity. In linear regression models, square root transformations were used where necessary to improve model residual distributions. The associations between IMR, RRR, or CFR with MVO and myocardial hemorrhage extent were assessed by Spearman rank correlation coefficients. Receiver operating characteristic curve analysis was performed to investigate the relationship between IMR, RRR, or CFR with MVO, and myocardial hemorrhage presence/absence, and clinical outcomes. Optimal predictive thresholds were derived from receiver operating characteristic curves. In this, sensitivity and specificity were considered equally important; therefore, the optimal cutoff was considered as the one giving the maximum Youden index. Receiver operating characteristic comparisons were made using the DeLong method. The incremental predictive ability of RRR was evaluated by calculating the continuous net reclassification improvement (NRI). Associations with heart failure hospitalizations, death/heart failure hospitalizations, or MACE were also evaluated using odds ratios (ORs), derived from logistic regression. All tests were 2-tailed, and a *P* value of <0.05 was considered statistically significant. There was no imputation for missing values. Statistical analyses were performed in SPSS (version 25.0, SPSS, IBM, Armonk, NY), MedCalc Statistical Software version 18 (MedCalc Software, Ostend, Belgium), or Rv3.2.4.

## Results

### Study Population Characteristics

**Figure 1.**
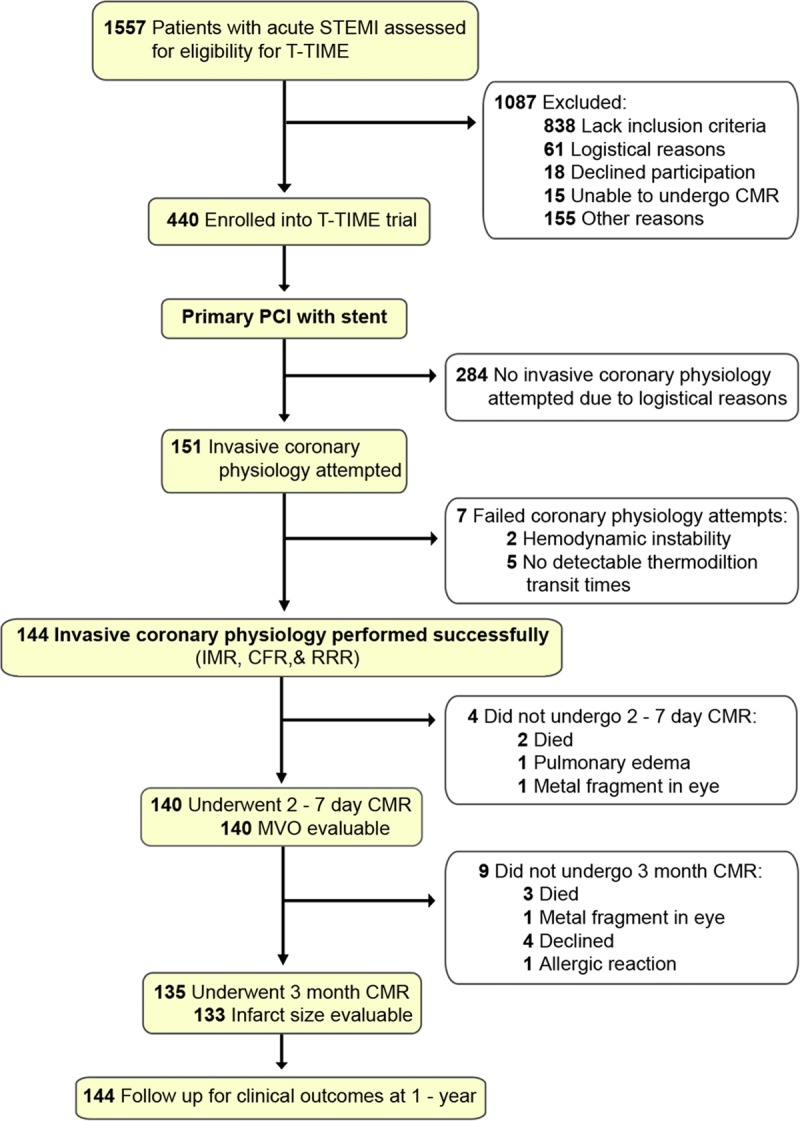
**Flow of subjects through the study.** CFR indicates coronary flow reserve; CMR, cardiovascular magnetic resonance; IMR, index of microcirculatory resistance; MVO, microvascular obstruction; PCI, percutaneous coronary intervention; RRR, resistive reserve ratio; and STEMI, ST-segment–elevation myocardial infarction.

The sample size (n=144) represented 33% of the overall T-TIME population and their characteristics were broadly similar (mean age 59±11 years, 80% male [Table 1, Figure 1]).

**Table 1.**
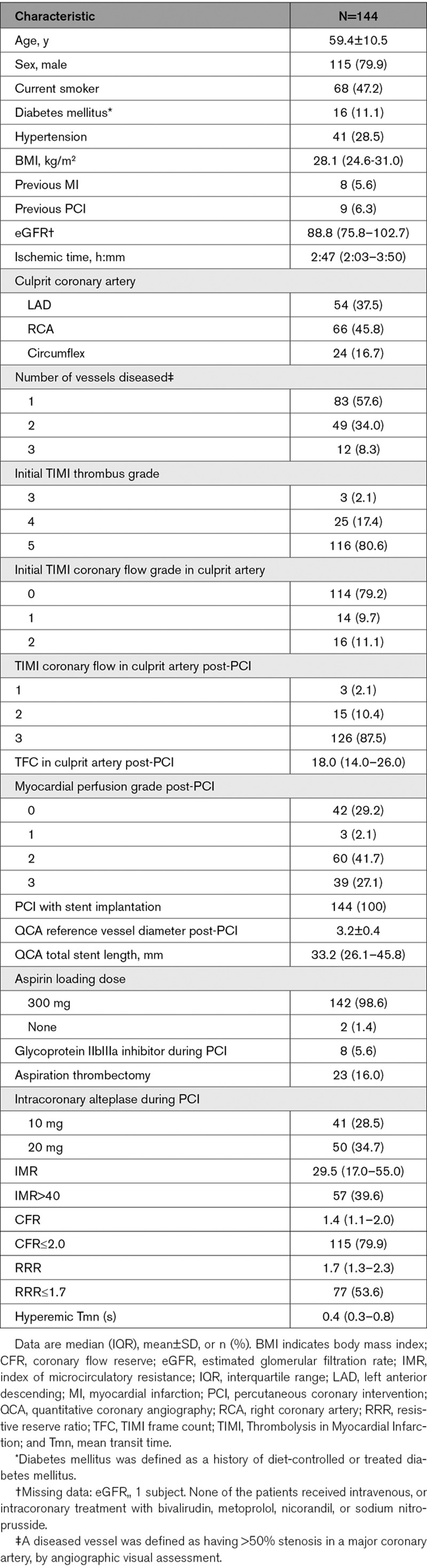
Population and Procedure Characteristics (n=144)

### Associations With Physiology Parameters

The characteristics that were associated with lower RRR, on multivariable linear regression, were: CFR≤2.0 (*P*<0.001) and IMR>40 (*P*=0.034). The characteristics that were associated with higher IMR were: higher TFC (*P*=0.015), MPG≤1 (*P*=0.017), and RRR≤1.7 (*P*=0.004). The only characteristic that was associated with lower CFR, on multivariable linear regression analysis, was RRR≤1.7 (*P*<0.001).

**Figure 2.**
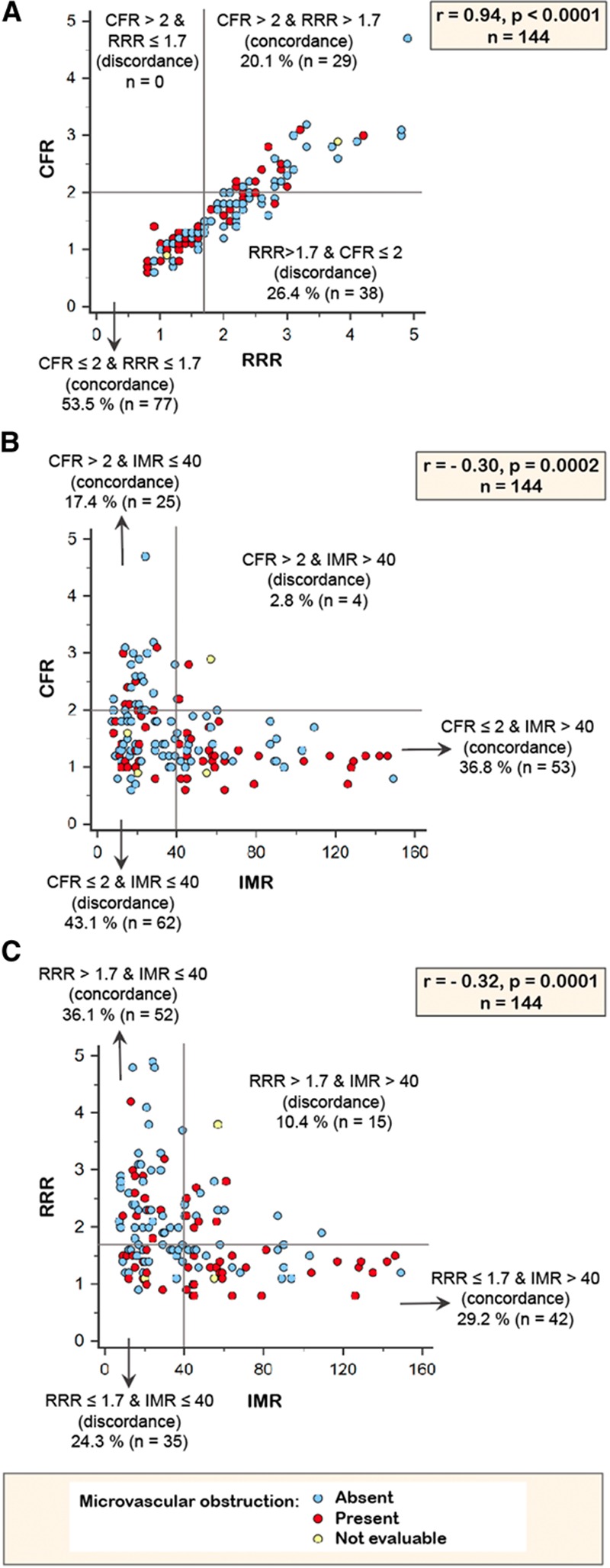
**Scatterplots showing correlations between coronary physiology parameters.** The following correlations are shown: (**A**) coronary flow reserve (CFR) and resistive reserve ratio (RRR); (**B**) index of microcirculatory resistance (IMR) and CFR; and (**C**) RRR and IMR. Also shown is discordance between dichotomized coronary physiology parameters and presence/absence of microvascular obstruction.

RRR was correlated with IMR (rho=−0.32; *P*=0.0001). RRR and CFR were correlated (rho=0.94, *P*<0.0001). CFR was correlated with IMR (rho=−0.30; *P*=0.0002) (Figure 2).

When CFR was dichotomized by 2, RRR by 1.7, and IMR by 40, discordance between CFR and RRR occurred in in 38 patients (26.4%), discordance between CFR and IMR occurred in 66 patients (45.9%), and discordance between RRR and IMR occurred in 50 patients (34.7%) (Figure 2).

### Associations of Physiology Parameters With CMR Characteristics

The CMR findings (Table I in the Data Supplement) reported below were broadly similar when the 18 patients with final TIMI coronary flow grades ≤2 were not included in the analyses (Tables II and III in the Data Supplement). Furthermore, the findings were broadly similar when RRR≤1.7 was substituted for RRR≤2.0 in multivariable regression analyses (Tables IV through VI and Figure I in the Data Supplement). Regression analyses using dichotomizations for IMR, CFR, and RRR according to optimal thresholds from the area under the curve (AUC) are also shown (Tables IV through VII in the Data Supplement).

### Relationships of Physiology Parameters With MVO

MVO was evaluable in 140 patients (97%). Given the high proportion of patients with a value of 0 for MVO (n=83 [59%]), the median MVO extent was 0.0 (interquartile range, 0.0–3.3). MVO was present in 57 patients (40.7%).

#### IMR

**Figure 3.**
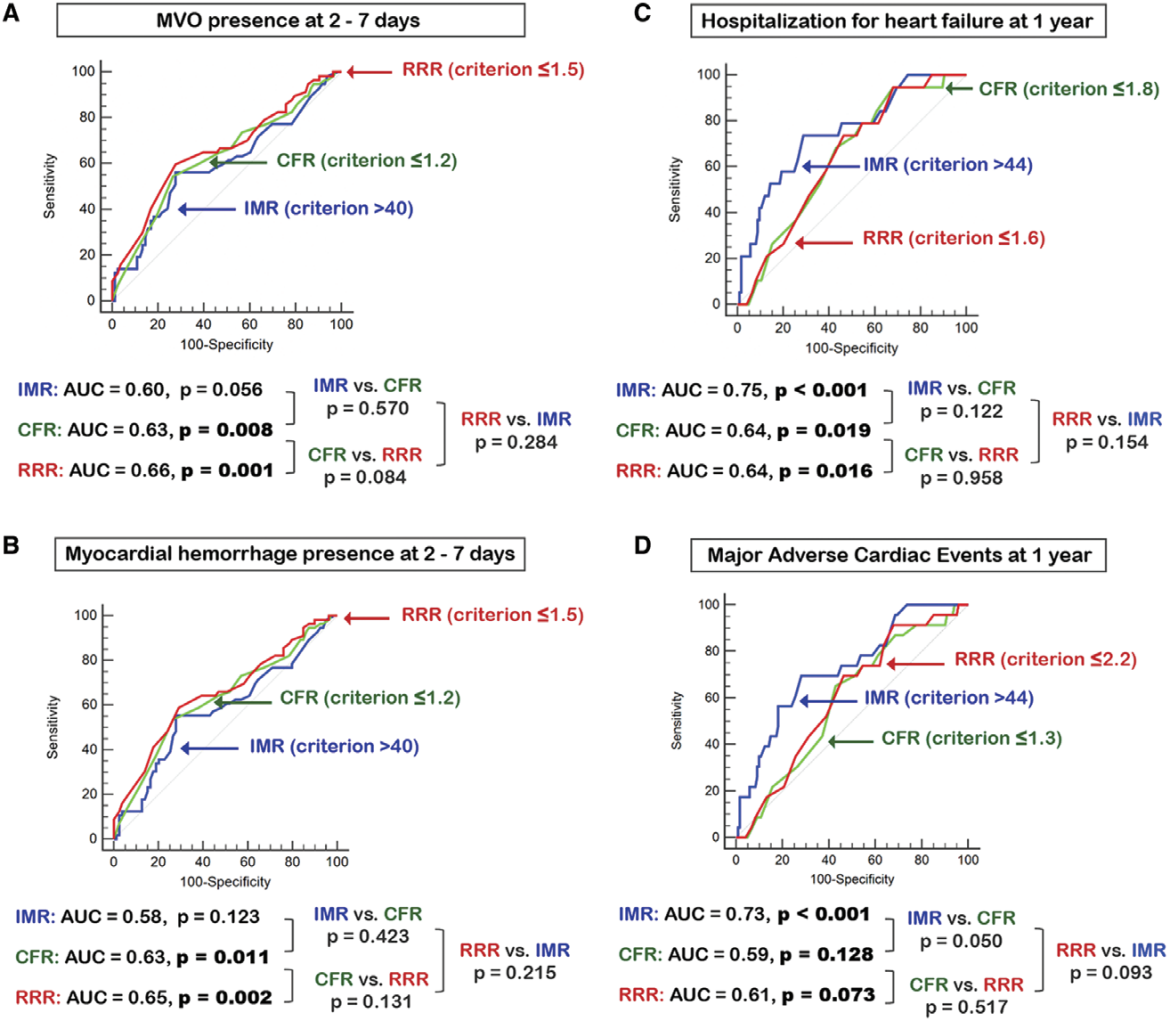
**DeLong comparisons of receiver operating characteristic curves, showing performance of index of microcirculatory resistance (IMR), coronary flow reserve (CFR), and resistive reserve ratio (RRR).** The predictive ability of IMR, CFR and RRR are shown for the following: **(A)** microvascular obstruction (MVO) presence/absence; **(B)** myocardial hemorrhage presence/absence; **(C)** hospitalization for heart failure; and **(D)** major adverse cardiac events.

Higher IMR measured acutely correlated with more MVO (rho=0.20, *P*=0.016; Figure II in the Data Supplement). IMR was >40 in 55 patients, of whom 32 (58.2%) had MVO present. The optimal IMR threshold from the AUC for predicting MVO presence was >40 (Figure 3). IMR>40 was multivariably associated with MVO extent and presence, independently of CFR≤2.0, RRR≤1.7, TFC, and MPG≤1, whereas continuous IMR was not (Table 2).

**Table 2.**
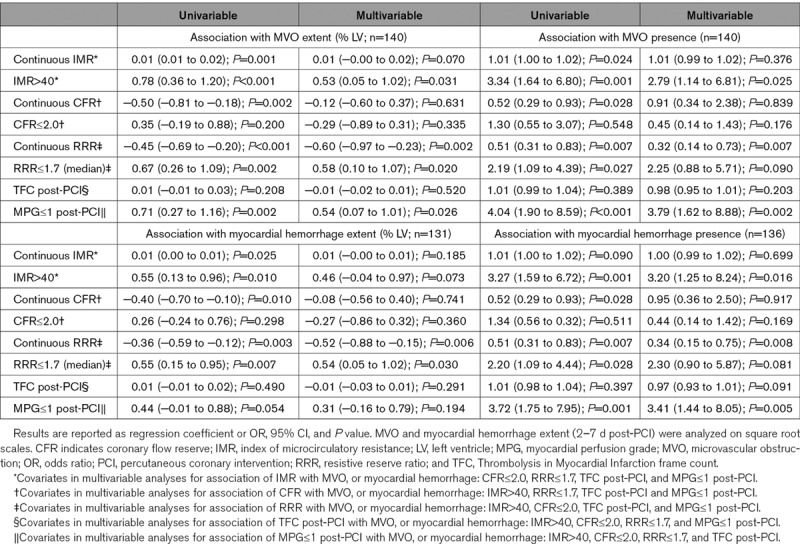
Associations of Coronary Physiology and Angiogram Parameters With MVO, or Myocardial Hemorrhage Extent, From Linear Regression and Their Associations With MVO, or Myocardial Hemorrhage, Presence From Logistic Regression

#### CFR

Lower CFR correlated with more MVO (rho=−0.27; *P*=0.001; Figure II in the Data Supplement). CFR was ≤2.0 in 112 patients, of whom 47 (42.1%) had MVO present. The optimal CFR threshold from the AUC for predicting MVO presence was ≤1.2 (Figure 3). Neither continuous CFR, nor CFR≤2.0, were associated with MVO (Table 2).

#### RRR

Lower RRR was correlated with more MVO (rho=−0.33; *P*=0.001; Figure II in the Data Supplement). RRR was ≤1.7 (median) in 75 patients, of whom 37 (49.3%) had MVO present. The optimal RRR threshold from the AUC for predicting MVO presence was ≤1.5 (Figure 3). Lower RRR was multivariably associated with MVO extent and presence, whereas RRR≤1.7 was associated with MVO extent, but not MVO presence (Table 2). The overall NRI, reflecting the incremental predictive accuracy for detecting the presence of MVO, was 0.66 (95% CI, 0.36–0.95; *P*<0.001) when RRR was added to a baseline model incorporating CFR≤2.0. When RRR≤1.7 was added to the baseline model containing CFR≤2.0, the NRI for detecting MVO presence was 0.34 (95% CI, 0.06–0.61; *P*=0.018). When the baseline model incorporated IMR>40, the overall NRI for detecting the presence of MVO was 0.38 (95% CI, 0.05–0.70; *P*=0.025) when RRR≤1.7 was added and was 0.29 ([95% CI, −0.02 to 0.59]; *P*=0.068) when RRR was added.

#### RRR and CFR in Combination

Compared with RRR>1.7 and CFR≤2.0 combined (reference group), the group with the combination of RRR≤1.7 and CFR≤2.0 was associated with an increased odds of MVO presence (37/75 [49.3%] versus 10/37 [27.0%]; OR, 2.63 [95% CI, 1.12–6.18]; *P*=0.027), and with more MVO (0.0 [0.0–5.3] versus 0.0 [0.0–0.8]; coefficient, 0.74; [95% CI, 0.22–1.25]; *P*=0.006).

### Relationships of Physiology Parameters With Myocardial Hemorrhage

Myocardial hemorrhage (the secondary manifestation of persistent MVO) occurred in 56 (41.2%) patients. Among those patients in whom myocardial hemorrhage was present, the median IMR was 41.5 (19.0–59.0), CFR was 1.2 (1.1–1.8), and RRR was 1.5 (1.2–2.2). Using multivariable logistic regression, patient and procedure characteristics that remained associated with the presence of myocardial hemorrhage were IMR>40 (*P*=0.034), MPG≤1 (*P*=0.006), and larger initial TIMI thrombus grade (*P*=0.033).

#### IMR

Myocardial hemorrhage occured in 31 (58.5%) patients with an IMR>40. The optimal IMR threshold for predicting myocardial hemorrhage presence was >40 (Figure 3). IMR>40 was multivariably associated with myocardial hemorrhage extent and presence, whereas continuous IMR was not (Table 2).

#### CFR

Lower CFR correlated with more myocardial hemorrhage (rho=−0.23, *P*=0.008) (Figure II in the Data Supplement). Myocardial hemorrhage was present in 46 (42.6%) patients with CFR≤2.0. The optimal CFR threshold for predicting myocardial hemorrhage presence was ≤1.2 (Figure 3). Neither continuous CFR, nor CFR≤2.0, were multivariable associates with myocardial hemorrhage (Table 2).

#### RRR

Lower RRR was correlated with more myocardial hemorrhage (rho=−0.28; *P*=0.001) (Figure II in the Data Supplement). Myocardial hemorrhage was present in 36 (50.0%) patients with RRR≤1.7. The optimal RRR threshold for predicting myocardial hemorrhage presence from the AUC was ≤1.5 (Figure 3). Lower RRR was multivariably associated with myocardial hemorrhage extent and presence, whereas RRR≤1.7 was associated with myocardial hemorrhage extent, but not its presence (Table 2). The overall NRI, reflecting the incremental predictive accuracy for detecting the presence of myocardial hemorrhage was 0.62 (95% CI, 0.32–0.91) *P*<0.001, when RRR was added to a baseline model incorporating CFR≤2.0. When RRR≤1.7 was added to the baseline model containing CFR≤2.0, the NRI for detecting myocardial hemorrhage presence was 0.34 ([95% CI, 0.05–0.62]; *P*=0.021). When the baseline model incorporated IMR>40, the overall NRI for detecting the presence of myocardial hemorrhage was 0.39 ([95% CI, 0.04–0.72]; *P*=0.026) when RRR≤1.7 was added, and was 0.25 ([95% CI, −0.07 to 0.56]; *P*=0.131) when RRR was added.

#### CFR and RRR in Combination

Compared with CFR≤2.0 and RRR>1.7 combined (reference group), the group with the combination of CFR≤2.0 and RRR≤1.7 was associated with an increased odds of myocardial hemorrhage presence (36/72 [50.0%] versus 10/36 [27.8%]; OR, 2.60; [95% CI, 1.10–6.17]; *P*=0.030), and with myocardial hemorrhage extent (0.0 [0.0–4.6] versus 0.0 [0.0–0.2]; coefficient, 0.62 [95% CI, 0.11–1.12]; *P*=0.017).

### Relationships of Physiology Parameters With 3-Month Infarct Size

Infarct size 3-month post-PCI was evaluable in 133 patients. The mean infarct size was 17.0±11.5%. Higher IMR was correlated with larger infarct size (rho=0.41; *P*<0.001) and IMR>40 was a multivariable associate of larger infarct size (Table 3).

**Table 3.**
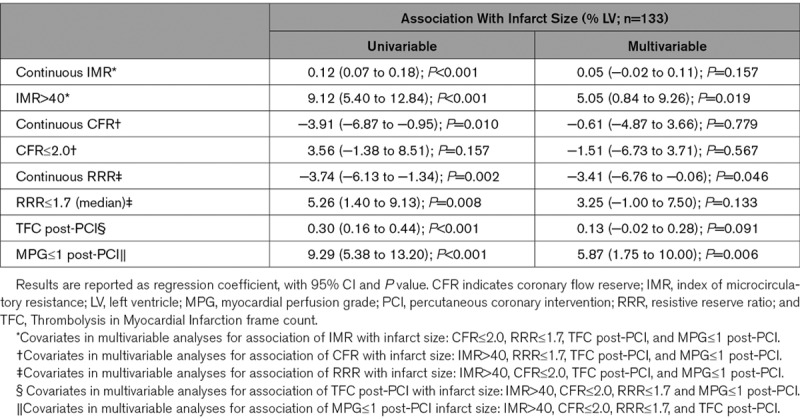
Associations of Coronary Physiology and Angiogram Parameters With Infarct Size, 3 Months Post-PCI, From Linear Regression

Lower CFR was correlated with larger infarct size (rho=−0.23; *P*=0.007), but there was no association when assessed by multivariable linear regression (Table 3).

Lower RRR was correlated with infarct size (rho=−0.22; *P*=0.012) and multivariably associated with larger infarct size (Table 3).

### Relationships of Physiology Parameters With Adjudicated Clinical Outcomes

At 1-year follow-up, there were 19 adjudicated hospitalizations for heart failure, 22 for all-cause death/heart failure hospitalization combined, and 23 MACE events.

#### IMR

In patients with IMR>40, heart failure hospitalizations occurred in 14 patients (24.6%) at 1 year, death/heart failure hospitalization occurred in 15 patients (26.3%), and MACE occurred in 16 (28.1%) patients. The optimal IMR threshold from the AUC for predicting heart failure hospitalization, or death/heart failure hospitalization was >44 (AUCs: 0.75 [*P*<0.001] and 0.72 [*P*<0.001], respectively). The optimal IMR threshold from the AUC for predicting MACE was also >44 (AUC: 0.73 [*P*<0.001]; Figure 3). Higher IMR was associated with heart failure hospitalizations, death/heart failure hospitalizations, and MACE (Table VII in the Data Supplement).

#### CFR

In those with CFR≤2.0, heart failure hospitalizations occurred in 18 patients (15.7%), death/heart failure hospitalization occurred in 20 patients (17.4%) and MACE occurred in 21 patients (18.3%). The optimal CFR threshold for predicting heart failure hospitalization was ≤1.8 (AUC: 0.64 [*P*=0.019]). The optimal CFR threshold for predicting death/heart failure hospitalization or MACE was ≤1.3 (AUCs: 0.63 [*P*=0.038] and 0.61 [*P*=0.073], respectively; Figure 3). Neither continuous CFR, CFR≤2.0, nor CFR≤1.4 (median) were associated with heart failure hospitalizations, death/heart failure hospitalization, or MACE (Table VII in the Data Supplement).

#### RRR

In those with RRR≤1.7, heart failure hospitalizations occurred in 14 (18.2%) patients, death/heart failure hospitalization occurred in 16 (20.8%) patients, and MACE occurred in 16 (20.8%) patients. The optimal RRR threshold for predicting heart failure hospitalization, or death/heart failure hospitalization was ≤1.6 (AUCs: 0.64 [*P*=0.016] and 0.62 [*P*=0.040], respectively). The optimal RRR threshold for predicting MACE was ≤2.2 (AUC: 0.59 [*P*=0.128]; Figure 3). Continuous RRR was associated with heart failure hospitalization, whereas RRR≤1.7 was not. RRR was not associated with death/heart failure hospitalization, or MACE (Table VII in the Data Supplement).

#### CFR and RRR in Combination

The combination of CFR≤2.0 and RRR≤1.7 did not enhance the prognostic significance of CFR≤2.0 and RRR>1.7 combined for association with heart failure hospitalization (14/77 [18.2%] versus 4/38 [10.5%]; OR, 1.89 [95% CI, 0.58–6.19]; *P*=0.294), death/heart failure hospitalization (16/77 [20.8] versus 4/38 [10.5]; OR, 2.23 [95% CI, 0.69–7.21]; *P*=0.180), or MACE (16/77 [20.8] versus 5/38 [13.2%]; OR, 1.73 [95% CI, 0.58–5.15]; *P*=0.324).

## Discussion

Our study provides new insights into the comparative clinical significance of invasive measures of microvascular function during primary PCI. Although CFR and RRR are correlated, we observed discordance between high and low dichotomized CFR and RRR values in 38 patients (26%), indicating that these parameters have overlapping and distinct behaviours. Furthermore, we observed differences in the associations of CFR and RRR, and their combination with MVO, myocardial hemorrhage, infarct size, and clinical outcomes, implying these tests do not have equivalent clinical significance.

IMR and RRR reflect different aspects of microvascular function and are complementary. IMR does not reflect microvascular vasodilator capacity. IMR may not reflect the full potential for the microcirculation to recover following reperfusion. RRR is a measure of the capacity of the coronary microcirculation to change from baseline to hyperemia, reflecting the ability to achieve maximal hyperemia.^14,15^ Lower RRR values indicate poor vasodilator capacity of the coronary microcirculation. Compared with CFR, RRR may better reflect the potential for the microcirculation to recover following reperfusion. RRR may provide additional information than what is obtained from currently available measures of microvascular function (CFR and IMR).

In prior studies, RRR was lower in patients with STEMI compared with non-STEMI and stable angina.^14^ Moreover, RRR measured in nonculprit vessels was lower acutely versus 1 month later, indicating a blunted hyperemic vasodilatory response acutely.^16^ In 45 acute STEMI patients, RRR≤1.98 (median for the cohort) measured post-primary PCI was associated with MVO extent 2 days post-PCI and infarct size at 6 months.^15^ However, to date, these findings have not been verified in a larger cohort and the association between RRR and clinical outcomes is unknown. Our study adds new data by (1) showing that RRR is associated with heart failure hospitalizations and (2) quantitatively comparing established coronary physiology parameters in both continuous and dichotomized form with the amount of MVO and hemorrhage.

Reliable identification of patients with high probability of having microvascular damage has potential to identify those patients in the catheterization laboratory for adjunctive therapies and inclusion in therapeutic trials. A test with a binary cutoff (normal/abnormal) is generally helpful for patient stratification. However, the optimal threshold may vary between different populations and different end points of interest. We chose established thresholds when dichotomizing IMR and CFR based on prior literature, that is, >40 and ≤2.0, respectively.^12^ There is no established threshold for RRR; therefore, the median was chosen when dichotomizing RRR. We observed discordance between dichotomized IMR>40 and MVO presence in just over one-third of patients (40%), which is similar to prior literature.^8^ Discordance was relatively higher between RRR≤1.7 and MVO presence (51%) and between CFR≤2.0 and MVO presence (58%). There are 2 explanations to consider. First, the extent of MVO varies in patients who have MVO present. Higher IMR and lower RRR or CFR are correlated with greater amounts of MVO, hence one would expect there to be discordance when binary thresholds are applied. Second, microvascular dysfunction is dynamic within minutes to the first few days post-reperfusion. When microvascular function is measured immediately post-primary PCI, reversible edema may contribute more to microvascular dysfunction, than on CMR 2 to 7 days later, where irreversible microvascular injury (including extravasation of red blood cells) may persist.

IMR>40 is being used to select patients for inclusion in clinical trials of adjunctive therapy during primary PCI, for example, pressure-controlled intermittent coronary sinus occlusion.^3,21^ Our study suggests that RRR may have potential as a superior tool compared with CFR, to guide patient selection for adjunctive therapy.

Although MPG≤1 post-PCI (but not TFC) was multivariably associated with more MVO, myocardial hemorrhage presence, and infarct size, and both MPG≤1, and TFC were associated with clinical outcomes, these parameters have several drawbacks. In particular, MPG is not quantitative, and the visual assessment of MPG has limited reproducibility.^22^ TFC provides a quantitative assessment of coronary blood flow, but it is confounded by nitrate use, heart rate, and the phase of the cardiac cycle in which dye is injected.^23^

### Limitations and Strengths

Strengths of our study include (1) multicenter enrolment, increasing generalizability, (2) blinding of coronary physiology measurements to minimize bias, (3) independent adjudication of clinical events, and (4) CMR was available in almost all patients (97%) at 2 to 7 days.

Limitations include the relatively small number of clinical events, which limited power to detect statistically significant associations. Since these were all emergency patients, they would not have withheld from caffeine, which could have affected response to adenosine and thus maximal hyperemia could not be guaranteed in all patients. However, these limitations apply to the previously published coronary physiology studies in the context of acute STEMI.^5,7,8,12^

Patients were not enrolled consecutively. Out of the 440 patients in the T-TIME trial, 284 patients had no invasive coronary physiology performed, and 7 patients had failed attempts at coronary physiology measurements (Figure 1). Typically, STEMI studies include a majority of left anterior descending artery infarcts. The reason for why invasive coronary physiology was most frequently performed in right coronary arteries is unclear, but one explanation may be logistical pressures for catheterization laboratory access given that it is typically easier to pass wires in the right coronary arteries than the left anterior descending.

### Conclusions

In patients with acute STEMI presenting within 6 hours of symptom onset, RRR and IMR were associated with MVO extent, myocardial hemorrhage presence, infarct size and clinical outcomes, whereas CFR was not. Compared with CFR, RRR seems to have superior potential for stratified therapy. More research is warranted.

## Acknowledgments

We thank the patients who participated in this study and the T-TIME investigators who supported it. We thank the members of the Trial Steering Committee, Data and Safety Monitoring Committee and Clinical Event Committee. We thank colleagues in the Robertson Centre for Biostatistics, University of Glasgow and the Glasgow Clinical Trials Unit.

## Sources of Funding

Dr Maznyczka is funded by a fellowship from the British Heart Foundation (FS/16/74/32573). Dr Berry is supported by grant RE/18/6/34217 and FS/16/74/32573 from the British Heart Foundation. T-TIME was supported by grant 12/170/4 from the Efficacy and Mechanism Evaluation (EME) programme of the National Institute for Health Research (NIHR-EME). Boehringer Ingelheim UK, Ltd provided the study drugs (alteplase 10 mg, 20 mg, and matched placebo). These organizations had no other involvement in the conduct of the study, or in any aspect of the manuscript. The research was in part supported by the NIHR infrastructure at Leeds.

## Disclosures

Dr Berry is employed by the University of Glasgow which holds research and/or consultancy agreements with AstraZeneca, Abbott Vascular, Boehringer Ingelheim, GSK, HeartFlow, Opsens, and Novartis. Dr Oldroyd has received speaker fees and research support from Abbott Vascular and Boston Scientific. Dr Cotton reported research support and speaker fees from Abbott Vascular. Dr Watkins reports speaker fees from Biosensors International, GE Healthcare, Abbott, Sanofi, and AstraZeneca.

## Supplementary Material


