# Supplementary material for: Comparative Significance of Invasive Measures of Microvascular Injury in Acute Myocardial Infarction
Source: Circ Cardiovasc Interv. 2020 May 15;13(5):e008505. doi: 10.1161/CIRCINTERVENTIONS.119.008505 (PMC7237023; doi:10.1161/CIRCINTERVENTIONS.119.008505)
Supplement: Supplementary file 1 [file hcv-13-e008505-s001.pdf]

SUPPLEMENTAL MATERIAL

Comparative Significance of Invasive Measures of Microvascular Injury in Acute Myocardial Infarction

ClinicalTrials.gov: NCT02257294

Contents

Eligibility Criteria Page 3

Angiogram Acquisition and Analysis Methods Page 6

Supplemental Table 1. CMR characteristics Page 9

Supplemental Table 2. From the 126 patients with final TIMI 3 coronary flow. Associations of coronary physiology parameters with MVO and myocardial hemorrhage Page 10

Supplemental Table 3. From the 126 patients with final TIMI 3 coronary flow. Associations of coronary physiology parameters with infarct size. Page 13

Supplemental Table 4. MVO and myocardial hemorrhage stratified according to IMR, RRR and CFR dichotomized by the optimal thresholds from the area under the curve (AUC); IMR, CFR and RRR dichotomized by their median values and; RRR dichotomized by 2. Page 15

Supplemental Table 5. Associations of coronary physiology parameters with MVO, or myocardial hemorrhage. Different dichotomizations for IMR, CFR and RRR are shown, including according to median, according to the optimal threshold from the AUC, and according to RRR≤2.0. Page 17

Supplemental Table 6. Associations of coronary physiology parameters with infarct size. Different dichotomizations for IMR, CFR and RRR are shown, including according to median, according to the optimal threshold from the AUC, and according to RRR≤2.0. Page 20

Supplemental Table 7. Associations between coronary physiology parameters and clinical outcomes at 1-year post-acute STEMI (n=144). Different dichotomizations for IMR, CFR and RRR are shown, including according to median, according to the optimal threshold from the AUC, and according to RRR≤2.0. Page 22

Supplemental Figure 1. Scatterplots showing correlations between **A**, coronary flow reserve (CFR) and RRR; and **B**, index of microcirculatory resistance (IMR) and RRR. Also shown is discordance between coronary physiology parameters (when CFR and RRR are dichotomized by 2) and presence/ absence of microvascular obstruction. Page 24

Supplemental Figure 2. Scatterplots showing correlations between IMR, RRR and CFR with microvascular obstruction (A, B and C) and with myocardial hemorrhage (D, E and F). Page 25

Eligibility Criteria

Patients with a clinical diagnosis of acute ST-segment elevation myocardial infarction (STEMI) were eligible for randomization according to the following eligibility criteria:

Inclusion

- Acute MI (symptom onset ≤ 6 hours) with persistent ST-segment elevation or recent left bundle branch block
- Coronary artery occlusion (TIMI [Thrombolysis in Myocardial Infarction] coronary flow grade 0 or 1), or impaired coronary flow (TIMI coronary flow grade 2, slow but complete filling) in the presence of definite angiographic evidence of thrombus (TIMI grade 2 or more)
- Proximal-mid culprit lesion location in a major coronary artery (i.e. the right, left anterior descending, intermediate, or circumflex artery)
- Radial artery access
- Successful coronary reperfusion (TIMI coronary flow grade ≥2) pre-stent achieved prior to randomization.
- Informed consent, i.e. only patients who were sufficiently well to understand the information about the study, as described by the attending cardiologist, were eligible to participate.

Exclusion

- Normal flow in the culprit coronary artery at initial angiography (TIMI grade 3)
- Functional coronary collateral supply (Rentrop grade 2/3) to the culprit artery
- Previous infarction in the culprit artery (known or suspected clinically, e.g. wall motion abnormality revealed by echocardiography
- Cardiogenic shock (Killip Class IV)
- Multivessel percutaneous coronary intervention (PCI) intended before the day 2-7 cardiovascular magnetic resonance (CMR) scan
- Estimated body weight <60 kg

- Non-cardiac co-morbidity with expected survival <1 year
- Contra-indication to contrast-enhance CMR imaging
- Pacemaker, or implantable defibrillator
- Known impaired renal function (estimated glomerular filtration rate <30ml/min)
- Significant bleeding disorder either at present or within the past 6 months
- Known hemorrhagic diathesis
- Patient with current concomitant oral anticoagulation therapy (international normalized ratio >1.3), including apixaban, dabigatran and rivaroxaban
- Any history of central nervous system damage (i.e. neoplasm, aneurysm, intracranial or spinal surgery)
- Severe hypertension (blood pressure >180/110 mmHg) not controlled by medical therapy
- Major surgery, biopsy of a parenchymal organ, or significant trauma within the past 3 months (this includes any trauma associated with the current acute MI)
- Recent trauma to the head (<2 months)
- Prolonged cardiopulmonary resuscitation (>2 minutes) within the past 2 weeks
- Acute pericarditis and/ or subacute bacterial endocarditis
- Acute pancreatitis
- Severe hepatic dysfunction, including hepatic failure, cirrhosis, portal hypertension (esophageal varices) and active hepatitis
- Active peptic ulceration
- Arterial aneurysm and known arterial/ venous malformation
- Neoplasm with increased bleeding risk
- Any known history of hemorrhagic stroke, or stroke of unknown origin
- Known history of ischemic stroke, or transient ischemic attack in the preceding 6 months
- Dementia

- Hypersensitivity to gentamicin, or natural rubber
- Incapacity, or inability to provide informed consent
- Previous randomization to this study, or participation in a study with an investigational drug, or medical device within 90 days prior to randomization
- Women of child bearing potential (i.e. pre-menopausal), or breast feeding
- Requirement for immunosuppressive therapy at any time during the preceding 3 months. This would include corticosteroids (but not inhaled or topical), drugs used following transplantation (e.g tacrolimus, cyclosporine), anti-metabolite therapies (e.g. mycophenolic acid, azathioprine, leflunomide and immunomodulators including biologics (e.g. adalimumab, or etanercept) and disease modifying anti-rheumatic drugs. This list is not exhaustive.
- Active or prophylactic treatment with oral, or parenteral antibiotic, antifungal, or antiviral therapy, to prevent or treat infection
- Any anti-cancer treatment (excluding surgery as this is covered above) at any time during the preceding 3 months, including chemotherapy, radiotherapy, and treatment with biologics, such as Vascular Endothelial Growth Factor Receptor (VEGFR) inhibitors (e.g. bevacizumab, pazopanib). This list is not exhaustive.
- Any significant concurrent, or recent condition(s) not listed above that in the opinion of the treating clinician would pose an additional risk to the patient.

Angiogram Acquisition and Analysis Methods

Coronary angiograms were acquired during emergency care with cardiac catheter laboratory X-ray and information technology equipment. The angiograms were analyzed using post-processing software (QAngio® XA Medis, Leiden, NL.) by experienced investigators who were blinded to treatment allocation. Catheter calibration was performed using the catheter calibration function on MEDIS QAngio. For each lesion, a view perpendicular to the long axis of the vessel was used in order to avoid foreshortening and overlap of branches. The single plane projection showing the best opacified and most severe lesion with minimal foreshortening and minimal branch overlap was selected. Feedback was provided to sites on the quality and completeness of the angiograms.

TIMI Coronary Flow Grade

The TIMI coronary flow grade was assessed using the following definitions[1]:

TIMI coronary flow grade	Definition
0	No flow
1	Minimal flow past obstruction
2	Slow (but complete) filling and slow clearance
3	Normal flow and clearance

TIMI Myocardial Perfusion Grade

TIMI myocardial perfusion grade provides a score for ground-glass appearance ('blush') of the contrast entering the microvasculature and contrast washout. TIMI myocardial perfusion grade was assessed according to the following definitions[2]:

TIMI myocardial perfusion grade	Definition
0	Minimal or no myocardial blush in the distribution of the culprit artery.
1	Myocardial blush is present in the distribution of the culprit artery. But there is incomplete clearance of dye between injections (with ~ 30 seconds between injections).
2	Myocardial blush is present in the distribution of the culprit artery. But there is slow contrast entry into the microvasculature and slow clearance of contrast. Specifically, blush is strongly persistent (i.e. either does not or only minimally diminishes in intensity) beyond 3 cardiac cycles after injection.
3	Myocardial blush is present in the distribution of the culprit artery, with normal entry and exit of dye (mild/ moderate persistence of dye beyond 3 cardiac cycles, but notably reduced after 3 cardiac cycles). Blush that is only mild intensity throughout 3 cardiac cycles after injection (washout phase), but fades minimally is also classified as grade 3.

TIMI Frame Count

The TIMI frame count represents the amount of time (in frames) for contrast dye to reach a standardized distal landmark[2]. If the culprit vessel was the left anterior descending artery the frame count was divided by 1.7 (correcting for longer vessel length).

TIMI Coronary Thrombus Grade

Thrombus burden revealed during coronary angiography was classified according to the TIMI thrombus grade[3]:

Thrombus grade	Definition
0	No angiographic characteristics of thrombus are present
1	Possible thrombus is present, with reduced contrast density, haziness, irregular lesion contour, or a smooth convex 'meniscus' at the site of total occlusion suggestive but not diagnostic of thrombus
2	Definite thrombus, with greatest dimensions ≤ half the vessel diameter
3	Definite thrombus but with greatest long axis dimension >1/2 but <2 vessel diameters
4	Definite thrombus, with the largest dimension ≥2 vessel diameters
5	Total occlusion

Supplemental Table 1. CMR characteristics**.** Results are median [IQR], mean ± SD, or n (%).

**Characteristic**	**CMR 2–7 days post-PCI (n=140)**	**CMR 3 months post-PCI (n=135)**
MVO present, n (%)*	57/140 (40.7)	-
MVO extent (% LV)*	0.0 [0.0-3.3]	-
Myocardial hemorrhage present, n (%)*	56/136 (41.2)	-
Myocardial hemorrhage extent (% LV)*	0.0 [0.0, 2.4]	-
Infarct size (% LV), mean ± SD*	24.1 ± 12.7	17.0 ± 11.5
LV ejection fraction (%), mean ± SD*	43.9 ± 8.3	49.1 ± 8.4
LV end diastolic volume (ml)*	166.0 [143.5-188.4]	162.8 [141.5-187.5]
LV end systolic volume (ml)*	90.5 [77.5-108.3]	81.1 [65.5-102.9]

* Missing data: Infarct size and LV (left ventricular) ejection fraction and volumes 2–7 days post-PCI (percutaneous coronary intervention), 4 subjects; MVO (microvascular obstruction) presence or absence/ extent, 4 subjects; myocardial hemorrhage extent, 13 subjects; myocardial hemorrhage presence or absence, 8 subjects; infarct size 3 months post-PCI, 11; LV ejection fraction and volumes 3 months post-PCI, 9; CMR, cardiovascular magnetic resonance; LV, left ventricle; PCI, percutaneous coronary intervention.

Supplemental Table 2. From the 126 patients with final TIMI 3 coronary flow. Associations of coronary physiology and angiogram parameters with MVO extent, or myocardial hemorrhage extent, from linear regression and their associations with MVO, or myocardial hemorrhage, presence (2-7days post-PCI) from logistic regression. Results are reported as regression coefficient or OR, 95% CI and p-value. MVO extent and myocardial hemorrhage extent (2-7 days post-PCI) were analyzed on square root scales.

	**Association with MVO extent (% LV) (n=123)**		**Association with MVO presence (n=123)**	
	**Univariable**	**Multivariable**	**Univariable**	**Multivariable**
**Continuous IMR***	0.24 (0.06, 0.41) **p=0.007**	0.01 (-0.00, 0.02) p=0.208	1.01 (1.00, 1.02) p=0.187	1.00 (0.99, 1.02) p=0.730
**IMR>40***	0.29 (0.11, 0.46) **p=0.001**	0.49 (-0.03, 1.02) **p=0.066**	3.14 (1.44, 6.87) **p=0.004**	2.76 (1.07, 7.15) **p=0.036**
**Continuous CFR†**	-0.25 (-0.43, -0.08) **p=0.005**	-0.20 (-0.69, 0.30) p=0.431	0.55 (0.30, 1.02) p=0.058	0.77 (0.27, 2.15) p=0.611
**CFR≤2.0†**	0.11 (-0.07, 0.29) p=0.234	-0.16 (-0.79, 0.46) p=0.601	1.27 (0.51, 3.14) p=0.604	0.61 (0.19, 1.99) p=0.410
**Continuous RRR‡**	-0.28 (-0.45, -0.11) **p=0.002**	-0.54 (-0.93, -0.16) **p=0.006**	0.55 (0.33, 0.92) **p=0.022**	0.39 (0.17, 0.91) **p=0.029**
**RRR≤1.7 (median)‡**	0.24 (0.06, 0.41) **p=0.008**	0.51 (0.00, 1.02) **p=0.049**	1.84 (0.88, 3.84) p=0.104	1.84 (0.70, 4.84) p=0.218
**TFC post-PCI §**	0.14 (-0.04, 0.32) p=0.131	0.01 (-0.03, 0.04) p=0.691	1.02 (0.96, 1.07) p=0.586	0.98 (0.92, 1.04) p=0.495
**MPG≤1 post-PCI ǁ**	0.25 (0.08, 0.43) **p=0.005**	0.60 (0.10, 1.11) **p=0.020**	4.64 (1.93, 11.18) **p=0.001**	4.17 (1.66, 10.45) **p=0.002**

	Association with myocardial hemorrhage extent (% LV) (n=131)		Association with myocardial hemorrhage presence (n=136)	
	**Univariable**	**Multivariable**	**Univariable**	**Multivariable**
**Continuous IMR***	0.01 (0.00, 0.02) **p=0.021**	0.01 (-0.00, 0.01) p=0.267	1.01 (1.00, 1.02) p=0.189	1.00 (0.99, 1.02) p=0.791
**IMR>40***	0.65 (0.18, 1.11) **p=0.007**	0.44 (-0.08, 0.97) p=0.099	3.50 (1.57, 7.80) **p=0.002**	3.07 (1.16, 8.14) **p=0.024**
**Continuous CFR†**	-0.40 (-0.72, -0.09) p**=0.014**	-0.14 (-0.62, 0.35) p=0.581	0.53 (0.29, 0.98) **p=0.044**	0.82 (0.30, 2.28) p=0.704
**CFR≤2.0†**	0.26 (-0.27, 0.79) p=0.329	-0.20 (-0.81, 0.41) p=0.522	1.34 (0.54, 3.32) p=0.527	0.59 (0.18, 1.95) p=0.389
**Continuous RRR‡**	-0.35 (-0.60, -0.10) **p=0.006**	-0.50 (-0.88, -0.12) **p=0.011**	0.54 (0.33, 0.90) **p=0.017**	0.40 (0.17, 0.92) **p=0.030**
**RRR≤1.7 (median)‡**	0.55 (0.12, 0.98) **p=0.013**	0.51 (0.01, 1.02) **p=0.048**	2.02 (0.96, 4.24) p=0.064	2.00 (0.76, 5.28) p=0.163
**TFC post-PCI §**	0.02 (-0.01, 0.05) p=0.197	0.01 (-0.03, 0.04) p=0.743	1.02 (0.96, 1.07) p=0.544	0.98 (0.92, 1.04) p=0.474
**MPG≤1 post-PCI‖**	0.51 (-0.00, 1.03) p=0.051	0.39 (-0.135, 0.90) p=0.140	4.43 (1.84, 10.68) **p=0.001**	3.87 (1.53, 9.80) **p=0.004**

* Covariates in multivariable analyses for association of IMR with MVO, or myocardial hemorrhage: CFR≤2.0, RRR≤1.7, TFC post-PCI and MPG≤1 post-PCI.

† Covariates in multivariable analyses for association of CFR with MVO, or myocardial hemorrhage: IMR>40, RRR≤1.7, TFC post-PCI and MPG≤1 post-PCI.

‡ Covariates in multivariable analyses for association of RRR with MVO, or myocardial hemorrhage: IMR>40, CFR≤2.0, TFC post-PCI and MPG≤1 post-PCI.

§ Covariates in multivariable analyses for association of TFC post-PCI with MVO, or myocardial hemorrhage: IMR>40, CFR≤2.0, RRR≤1.7 and MPG≤1 post-PCI.

|| Covariates in multivariable analyses for association of MPG≤1 post-PCI with MVO, or myocardial hemorrhage: IMR>40, CFR≤2.0, RRR≤1.7 and TFC post-PCI.

CFR, Coronary flow reserve; IMR, index of microcirculatory resistance; LV, left ventricle; MPG, myocardial perfusion grade; MVO, microvascular obstruction; RRR, resistive reserve ratio; TFC, TIMI frame count.

Supplemental Table 3. From the 126 patients with final TIMI 3 coronary flow. Associations of coronary physiology parameters with infarct size, 3 months post-PCI, from linear regression. Results are reported as regression coefficient, with 95% confidence interval and p-value.

	**Association with infarct size (% LV) (n=117)**	
	**Univariable**	**Multivariable**
**Continuous IMR***	0.11 (0.04, 0.18) **p=0.002**	0.06 (-0.02, 0.14) p=0.130
**IMR>40***	7.75 (3.78, 11.72) **p<0.001**	5.12 (0.62, 9.61) **p=0.026**
**Continuous CFR†**	-3.30(-6.20, -0.41) **p=0.026**	-0.86 (-5.15, 3.42) p=0.691
**CFR≤2.0†**	2.37 (-2.44, 7.17) p=0.331	-1.29 (-6.58, 3.99) p=0.629
**Continuous RRR‡**	-2.90(-5.25, -0.55) **p=0.016**	-2.84 (-6.28, 0.60) p=0.104
**RRR≤1.7 (median)‡**	4.10 (0.22, 7.99) **p=0.038**	2.91 (-1.47, 7.29) p=0.190
**TFC post-PCI §**	0.37 (0.08, 0.67) **p=0.013**	0.17 (-0.13, 0.47) p=0.267
**MPG≤1 post-PCI ||**	7.34 (2.96, 11.71) **p=0.001**	5.97 (1.69, 10.25) **p=0.007**

* Covariates in multivariable analyses for association of IMR with MVO, or myocardial hemorrhage: CFR≤2.0, RRR≤1.7, TFC post-PCI and MPG**≤1** post-PCI.

† Covariates in multivariable analyses for association of CFR with MVO, or myocardial hemorrhage: IMR>40, RRR≤1.7, TFC post-PCI and MPG≤1 post-PCI.

‡ Covariates in multivariable analyses for association of RRR with MVO, or myocardial hemorrhage: IMR>40, CFR≤2.0, TFC post-PCI and MPG≤1 post-PCI.

§ Covariates in multivariable analyses for association of TFC post-PCI with MVO, or myocardial hemorrhage: IMR>40, CFR≤2.0, RRR≤1.7 and MPG≤1 post-PCI.

|| Covariates in multivariable analyses for association of MPG≤1 post-PCI with MVO, or myocardial hemorrhage: IMR>40, CFR≤2.0, RRR≤1.7 and TFC post-PCI.

CFR, Coronary flow reserve; IMR, index of microcirculatory resistance; LV, left ventricle; MPG, myocardial perfusion grade; MVO, microvascular obstruction; RRR, resistive reserve ratio; TFC, TIMI frame count

Supplemental Table 4. MVO and myocardial hemorrhage stratified according to IMR, RRR and CFR dichotomized by the optimal thresholds from the area under the curve (AUC); IMR, CFR and RRR dichotomized by their median values and; RRR dichotomized by 2. Results are median [IQR], mean ± SD, or n (%).

	MVO extent (% LV) 2-7 days post-PCI (n=140)	MVO presence 2-7 days post-PCI (n=140)	Myocardial hemorrhage extent (% LV) 2-7 days post-PCI (n=131)	Myocardial hemorrhage presence 2-7 days post-PCI (n=136)	Infarct size (% LV) 3 months post-PCI (n=133)
**IMR dichotomized by optimal threshold from AUC**					
**≤40 (n=57)**	0.0 [0.0-1.1]	25/85 (29.4)	0.0 [0.0-0.6]	25/83 (30.1)	13.4 ± 10.3
**>40 (n=87)**	1.8 [0.0-5.2]	32/55 (58.2)	0.5 [0.0-4.4]	31/53 (58.5)	22.5 ± 11.0
**IMR dichotomized by median**					
**≤30 (n=69)**	0.0 [0.0-2.8]	25/73 (34.2)	0.0 [0.0-2.3]	25/71 (35.2)	13.2 ± 10.6
**>30 (n=75)**	0.0 [0.0-4.5]	32/67 (47.8)	0.0 [0.0-2.6]	31/65 (47.7)	21.3 ± 11.0
**CFR dichotomized by optimal threshold from AUC**					
**≤1.2 (n=55)**	1.8 [0.0-6.2]	31/53 (58.5)	0.5 [0.0-4.8]	30/51 (58.8)	19.9 ± 11.2
**>1.2 (n=89)**	0.0 [0.0-1.0]	26/87 (29.9)	0.0 [0.0-0.7]	26/85 (30.6)	15.3 ± 11.4
**CFR dichotomized by median**					
**≤1.4 (n=78)**	0.0 [0.0-5.3]	37/76 (48.7)	0.0 [0.0-4.5]	36/73 (49.3)	19.2 ± 12.2

**>1.4 (n=66)**	0.0 [0.0-1.0]	20/64 (31.3)	0.0 [0.0-0.6]	20/63 (31.7)	14.5 ± 10.1
**CFR dichotomized by2.0**					
**≤2.0 (n=115)**	0.0 [0.0-3.4]	47/112 (42.0)	0.0 [0.0-2.4]	46/108 (42.6)	17.7 ± 11.6
**>2.0 (n=29)**	0.0 [0.0-3.0]	10/28 (35.7)	0.0 [0.0-2.1]	10/28 (35.7)	14.2 ± 10.7
**RRR dichotomized by optimal threshold from AUC**					
**≤1.5 (n=59)**	2.5 [0.0-7.2]	34/57 (59.6)	0.9 [0.0-4.9]	33/56 (58.9)	20.2 ± 12.4
**>1.5 (n=85)**	0.0 [0.0-0.8]	23/83 (27.7)	0.0 [0.0-0.5]	23/80 (28.8)	14.8 ± 10.3
**RRR dichotomized by median**					
**≤1.7 (n=77)**	0.0 [0.0-5.3]	37/75 (49.3)	0.0 [0.0-4.6]	36/72 (50.0)	19.5 ± 12.0
**>1.7 (n=67)**	0.0 [0.0-0.9]	20/65 (30.8)	0.0 [0.0-0.6]	20/64 (31.3)	14.2 ± 10.2
**RRR dichotomized by 2.0**					
**≤2.0 (n=92)**	0.0 [0.0-5.0]	40/89 (44.9)	0.0 [0.0-4.2]	39/85 (45.9)	18.2 ± 12.0
**>2.0 (n=52)**	0.0 [0.0-1.0]	17/51 (33.3)	0.0 [0.0-0.6]	17/51 (33.3)	14.9 ± 10.3

AUC, area under the curve; LV, left ventricle; MVO, microvascular obstruction; PCI, percutaneous coronary intervention; RRR, resistive reserve ratio.

Supplemental Table 5. Associations of coronary physiology and angiogram parameters with microvascular obstruction extent, or myocardial hemorrhage extent from linear regression and their associations with microvascular obstruction, or myocardial hemorrhage, presence from logistic regression. Different dichotomizations for IMR, CFR and RRR are shown, including according to median values and according to the optimal thresholds from AUCs. Results are reported as regression coefficient or odds ratio, with 95% confidence interval and p-value. Microvascular obstruction extent and myocardial haemorrhage extent (2-7 days post-PCI) were analysed on square root scales.

	**Association with MVO extent (% LV) (n=140)**		**Association with MVO presence (n=140)**	
	**Univariable**	**Multivariable**	**Univariable**	**Multivariable**
**Continuous IMR***	0.01 (0.01, 0.02) **p=0.001**	0.01 (0.00, 0.02) **p=0.027**	1.01 (1.00, 1.02) **p=0.024**	1.01 (1.00, 1.02) **p=0.199**
**IMR>40 (optimal threshold from AUC)***	0.78 (0.36, 1.20) **p<0.001**	0.65 (0.17, 1.12) **p=0.008**	3.34 (1.64, 6.80) **p=0.001**	3.18 (1.33,7.59) **p=0.009**
**IMR>30 (median)***	0.43 (0.01, 0.85) **p=0.045**	0.21 (-0.26, 0.68) p=0.384	1.76 (0.89, 3.47) p=0.105	1.37 (0.61, 3.11) p=0.449
**Continuous CFR†**	-0.50(-0.81, -0.18) **p=0.002**	-0.24 (-0.74, 0.26) p=0.344	0.52 (0.29, 0.93) **p=0.028**	0.58 (0.20, 1.63) p=0.297
**CFR≤2.0†**	0.35 (-0.19, 0.88) p=0.200	-0.40 (-1.08, 0.29) p=0.252	1.30 (0.55, 3.07) p=0.548	0.47 (0.13, 1.74) p=0.473
**CFR≤1.2 (optimal threshold from AUC)†**	0.85 (0.44, 1.27) **p<0.001**	0.65 (0.14, 1.17) **p=0.014**	3.31 (1.62, 6.75) **p=0.001**	3.68 (1.36, 9.98) **p=0.010**
**CFR≤1.4 (median)†**	0.65 (0.23, 1.06) **p=0.002**	0.50 (-0.18, 1.17) p=0.151	2.09 (1.04, 4.18) **p=0.038**	2.72 (0.73, 10.21) p=0.137
**Continuous RRR‡**	-0.45 (-0.69, -0.20) **p<0.001**	-0.60 (-0.97, -0.23) **p=0.002**	0.51 (0.31, 0.83) **p=0.007**	0.32 (0.14, 0.73) **p=0.007**
**RRR≤2.0‡**	0.56 (0.13, 0.99) **p=0.012**	0.58 (0.02, 1.14) **p=0.043**	2.19 (1.09, 4.39) **p=0.027**	1.79 (0.62, 5.20) p=0.286
**RRR≤1.5 (optimal threshold from AUC)‡**	0.98 (0.57, 1.38) **p<0.001**	0.90 (0.46, 1.35) **p<0.001**	3.86 (1.89, 7.88) **p<0.001**	4.63 (1.88, 11.40) **p=0.001**
**RRR≤1.7 (median)†**	0.67 (0.26, 1.09) **p=0.002**	0.58 (0.10, 1.07) **p=0.020**	2.19 (1.09, 4.39) **p=0.027**	0.25 (0.88, 5.71) p=0.090

**TFC post-PCI §**	0.01 (-0.01, 0.03) p=0.208	-0.01 (-0.03, 0.01) p=0.393	1.01 (0.99, 1.04) p=0.389	0.98 (0.65, 1.01) p=0.172
**MPG≤1 post-PCI ‖**	0.71 (0.27, 1.16) **p=0.002**	0.52 (0.04, 0.99) **p=0.034**	4.04 (1.90, 8.59) **p<0.001**	3.64 (1.57, 8.43) **p=0.003**
	**Association with myocardial hemorrhage extent (% LV) (n=131)**		**Association with myocardial hemorrhage presence (n=136)**	
	**Univariable**	**Multivariable**	**Univariable**	**Multivariable**
**Continuous IMR***	0.01 (0.00, 0.01) **p=0.025**	0.01 (-0.00, 0.01) p=0.120	1.01 (1.00, 1.02) p=0.090	1.01 (0.99, 1.02) p=0.508
**IMR>40 (optimal threshold from AUC)***	0.55 (0.13, 0.96) **p=0.010**	0.55 (0.04, 1.05) **p=0.034**	3.27 (1.59, 6.72) **p=0.001**	3.57 (1.40, 9.09) **p=0.008**
**IMR>30 (median)***	0.23 (-0.18, 0.64) p=0.278	0.07 (-0.43, 0.57) p=0.776	1.68 (0.84, 3.34) p=0.141	1.32 (0.54, 3.22) p=0.539
**Continuous CFR†**	-0.40 (-0.70, -0.10) p=**0.010**	-0.17 (-0.66, 0.32) p=0.496	0.52 (0.29, 0.93) **p=0.028**	0.63 (0.22, 1.75) p=0.371
**CFR≤2.0†**	0.26 (-0.24, 0.76) p=0.298	-0.39 (-1.06, 0.29) p=0.258	1.34 (0.56, 0.32) p=0.511	0.46 (0.12, 1.68) p=0.237
**CFR≤1.2 (optimal threshold from AUC)†**	0.65 (0.24, 1.07) **p=0.002**	0. 47 (-0.06, 0.99) p=0.079	3.24 (1.57, 6.69) **p=0.001**	3.39 (1.24, 9.26) **p=0.018**
**CFR≤1.4 (median)†**	0.53 (0.13, 0.93) **p=0.010**	0.40 (-0.28, 1.07) p=0.245	2.09 (1.04, 4.22) **p=0.039**	2.66 (0.71, 9.98) p=0.148
**Continuous RRR‡**	-0.36 (-0.59, -0.12) **p=0.003**	-0.52 (-0.88, -0.15) **p=0.006**	0.51 (0.31, 0.83) **p=0.007**	0.34 (0.15, 0.75) **p=0.008**
**RRR≤2.0‡**	0.48 (0.06, 0.89) **p=0.025**	0.56 (0.01, 1.11) **p=0.048**	1.70 (0.82, 3.49) p=0.152	1.88 (0.65, 5.45) p=0.245
**RRR≤1.5 (optimal threshold from AUC)‡**	0.78 (0.38 1.18) **p<0.001**	0.76 (0.31, 1.22) **p=0.001**	3.56 (1.73, 7.30) **p=0.001**	4.10 (1.65, 10.19) **p=0.002**
**RRR≤1.7 (median)†**	0.55 (0.15, 0.95) **p=0.007**	0.54 (0.05, 1.02) **p=0.030**	2.20 (1.09, 4.44) **p=0.028**	2.30 (0.90, 5.87) p=0.081
**TFC post-PCI §**	0.01 (-0.01, 0.02) p=0.490	-0.11 (-0.32, 0.10) p=0.292	1.01 (0.98, 1.04) p=0.397	0.97 (0.93, 1.01) p=0.099
**MPG≤1 post-PCI ‖**	0.44 (-0.01, 0.88) p=0.054	0.29 (-0.19, 0.77) p=0.236	3.72 (1.75, 7.95) **p=0.001**	3.27 (1.40, 7.65) **p=0.006**

* Covariates in multivariable analyses for association of IMR with MVO, or myocardial hemorrhage: CFR≤2.0, RRR≤2.0, TFC post-PCI and MPG**≤1** post-PCI.

† Covariates in multivariable analyses for association of CFR with MVO, or myocardial hemorrhage: IMR>40, RRR≤2.0, TFC post-PCI and MPG≤1 post-PCI.

‡ Covariates in multivariable analyses for association of RRR with MVO, or myocardial hemorrhage: IMR>40, CFR≤2.0, TFC post-PCI and MPG≤1 post-PCI.

§ Covariates in multivariable analyses for association of TFC post-PCI with MVO, or myocardial hemorrhage: IMR>40, CFR≤2.0, RRR≤2.0 and MPG≤1 post-PCI.

|| Covariates in multivariable analyses for association of MPG≤1 post-PCI with MVO, or myocardial hemorrhage: IMR>40, CFR≤2.0, RRR≤2.0 and TFC post-PCI.

CFR, Coronary flow reserve; IMR, index of microcirculatory resistance; LV, left ventricle; MPG, myocardial perfusion grade; MVO, microvascular obstruction; RRR, resistive reserve ratio; TFC, TIMI frame count.

Supplemental Table 6. Associations of coronary physiology and angiogram parameters with infarct size, 3 months post-PCI, from linear regression. Different dichotomizations for IMR, CFR and RRR are shown, including according to median values and according to the optimal thresholds from AUCs. Results are reported as regression coefficient, with 95% confidence interval and p-value.

	**Association with infarct size (% LV) (n=133)**	
	**Univariable**	**Multivariable**
**Continuous IMR***	0.12 (0.07, 0.18) **p<0.001**	0.06 (-0.00, 0.13) p=0.065
**IMR>40 (optimal threshold from AUC)****	9.12 (5.40, 12.84) **p<0.001**	5.77 (1.63, 9.91) **p=0.007**
**IMR>30 (median)***	8.01 (4.30, 11.72) **p<0.001**	4.83 (0.77, 0.89) **p=0.020**
**Continuous CFR†**	-3.91 (-6.87, -0.95) **p=0.026**	-3.37 (-7.63, 0.90) p=0.120
**CFR≤2.0†**	3.56 (-1.38, 8.51) p=0.157	0.11 (-5.83, 6.05) p=0.971
**CFR≤1.2 (optimal threshold from AUC)†**	4.61 (0.61, 8.61) **p=0.024**	2.09 (-2.45, 6.64) p=0.364
**CFR≤1.4 (median)†**	4.75 (0.86, 8.63) **p=0.017**	5.19 (-0.58, 10.95) p=0.077
**Continuous RRR‡**	-3.74 (-6.13, -1.34) **p=0.002**	-3.41 (-6.76, -0.06) **p=0.046**
**RRR≤2.0 ‡**	3.27 (-0.81, 7.35) p=0.115	0.41 (-4.47, 5.29) p=0.869
**RRR≤1.5 (optimal threshold from AUC)‡**	5.47 (1.57, 9.37) **p=0.006**	3.28 (-0.73, 7.29) p=0.108
**RRR≤1.7 (median)‡**	5.26 (1.40, 9.13) **p=0.008**	3.25 (-1.00, 7.50) p=0.133
**TFC post-PCI §**	0.30 (0.16, 0.44) **p<0.001**	0.13 (-0.02, 0.28) p=0.098
**MPG≤1 post-PCI ‖**	9.29 (5.38, 13.20) **p<0.001**	5.87 (1.73, 10.01) **p=0.006**

* Covariates in multivariable analyses for association of IMR with MVO, or myocardial hemorrhage: CFR≤2.0, RRR≤2.0, TFC post-PCI and MPG**≤1** post-PCI.

† Covariates in multivariable analyses for association of CFR with MVO, or myocardial hemorrhage: IMR>40, RRR≤2.0, TFC post-PCI and MPG≤1 post-PCI.

‡ Covariates in multivariable analyses for association of RRR with MVO, or myocardial hemorrhage: IMR>40, CFR≤2.0, TFC post-PCI and MPG≤1 post-PCI.

§ Covariates in multivariable analyses for association of TFC post-PCI with MVO, or myocardial hemorrhage: IMR>40, CFR≤2.0, RRR≤2.0 and MPG≤1 post-PCI.

|| Covariates in multivariable analyses for association of MPG≤1 post-PCI with MVO, or myocardial hemorrhage: IMR>40, CFR≤2.0, RRR≤1.7 and TFC post-PCI.

CFR, Coronary flow reserve; IMR, index of microcirculatory resistance; LV, left ventricle; MPG, myocardial perfusion grade; MVO, microvascular obstruction; RRR, resistive reserve ratio; TFC, TIMI frame count

Supplemental Table 7. Unadjusted odds ratios and 95% confidence intervals, from logistic regression, showing associations of coronary physiology and angiogram parameters with clinical outcomes at 1-year post-acute STEMI (n=144). Different dichotomizations for IMR, CFR and RRR are shown, including according to median values and according to the optimal thresholds from AUCs.

	**Hospitalization for heart failure (n=19 events)**		**All-cause death & heart failure hospitalization combined (22 events)**		**Major adverse cardiac events (n=23 events)**	
	**Crude OR (95% CI)**	**p-value**	**Crude OR (95% CI)**	**p-value**	**Crude OR (95% CI)**	**p-value**
**Continuous IMR**	1.02 (1.01, 1.04)	**<0.001**	1.02 (1.01, 1.03)	**0.001**	1.02 (1.01, 1.03)	**0.001**
**IMR>40**	5.34 (1.80, 15.81)	**0.002**	4.08 (1.55, 10.79)	**0.005**	4.46 (1.70, 11.70)	**0.002**
**IMR>44 (optimal threshold from AUC)**	6.92 (2.32, 20.63)	**0.001**	5.33 (2.00, 14.18)	**0.001**	5.85 (2.21, 15.47)	**<0.001**
**IMR>30 (median)**	3.56 (1.21, 10.50)	**0.021**	2.70 (1.03, 7.09)	**0.044**	2.93 (1.13, 7.64)	**0.028**
**Continuous CFR**	0.39 (0.15, 1.04)	0.060	0.48 (0.20, 1.14)	0.097	0.57 (0.26, 1.28)	0.174
**CFR≤2.0**	5.20 (0.66, 40.65)	0.116	0.35 (0.08, 1.60)	0.177	3.02 (0.67, 13.68)	0.152
**CFR≤ optimal threshold from AUC***	8.47 (1.09, 65.70)	**0.041**	2.89 (1.10, 7.58)	**0.032**	2.49 (0.98, 6.31)	0.055
**CFR≤1.4 (median)**	2.67 (0.91, 7.86)	0.075	2.58 (0.95, 7.04)	0.064	2.18 (0.84, 5.66)	0.112
**Continuous RRR**	0.44 (0.19, 0.99)	**0.047**	0.55 (0.28, 1.10)	0.092	0.59 (0.31, 1.14)	0.119
**RRR≤2.0**	2.34 (0.73, 7.46)	0.151	2.12 (0.74, 6.16)	0.163	1.74 (0.64, 4.73)	0.279
**RRR≤ optimal threshold from AUC†**	3.23 (1.10, 9.52)	**0.033**	3.14 (1.15, 8.57)	**0.025**	4.99 (1.12, 22.37)	**0.036**

**RRR≤1.7 (median)**	2.76 (0.94, 8.11)	0.066	2.67 (0.98, 7.27)	0.055	2.25 (0.86, 5.85)	0.097
**TFC post-PCI**	1.04 (1.01, 1.07)	**0.007**	1.05 (1.02, 1.09)	**0.002**	1.05 (1.02, 1.08)	**0.002**
**MPG≤1 post-PCI**	4.78 (1.74, 13.17)	**0.002**	3.24 (1.28, 8.20)	**0.013**	2.91 (1.17, 7.23)	**0.022**

*CFR≤1.8 was the optimal threshold from AUC for predicting heart failure hospitalization; CFR≤1.3 was the optimal threshold from AUC for all-cause death and heart failure hospitalization; CFR≤1.3 was the optimal threshold from AUC for major adverse cardiac events.

†RRR≤1.6 was the optimal threshold from AUC for predicting heart failure hospitalization; CFR≤1.6 was optimal threshold from AUC for predicting all cause death of heart failure hospitalization; CFR≤2.2 was the optimal threshold for predicting major adverse cardiac events.

AUC, area under the curve; CFR, Coronary flow reserve; IMR, index of microcirculatory resistance; RRR, resistive reserve ratio.

Supplemental Figure 1.

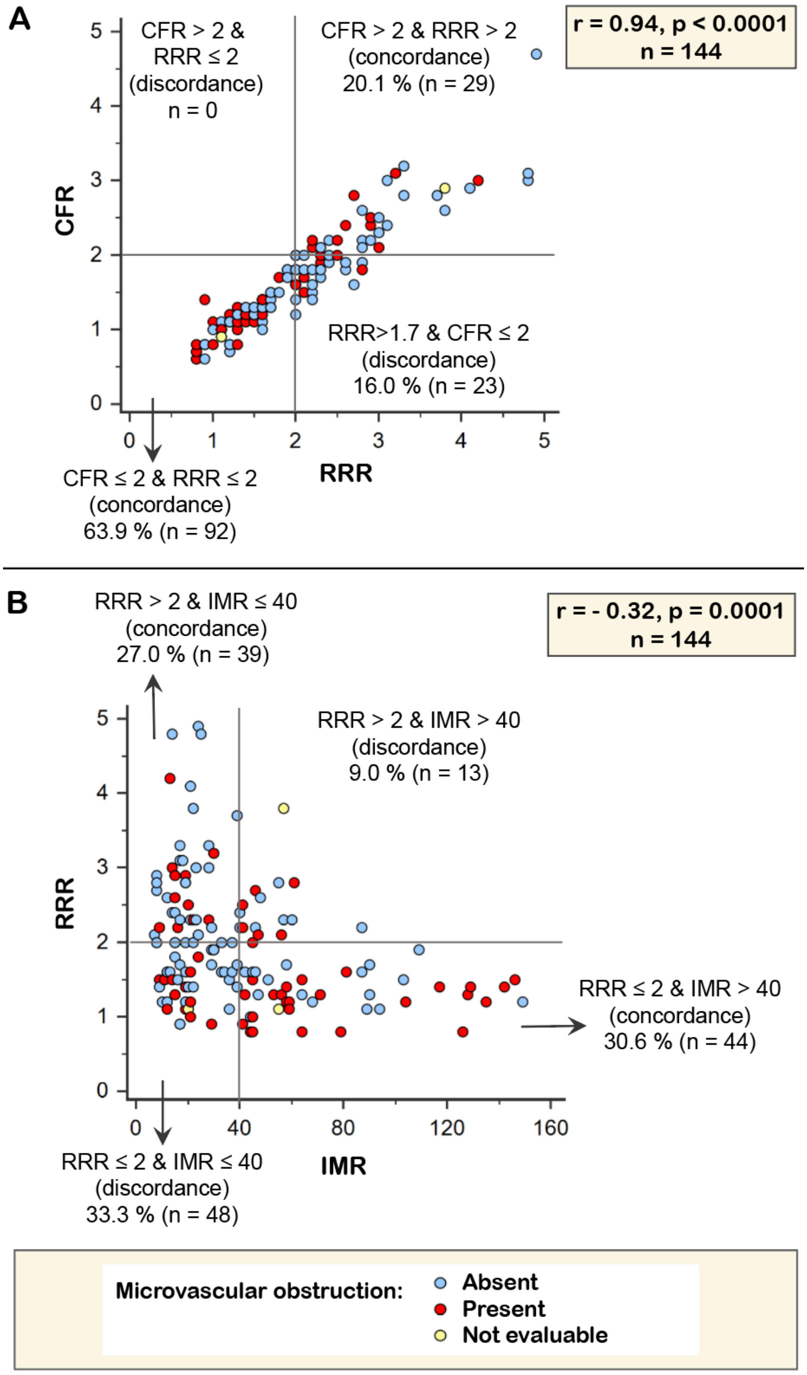

Scatterplots showing correlations between A, coronary flow reserve (CFR) and resistive reserve ratio (RRR); and B, index of microcirculatory resistance (IMR) and RRR. Also shown is discordance between coronary physiology parameters (when CFR and RRR are dichotomized by 2) and presence/ absence of microvascular obstruction.

Supplemental Figure 2.

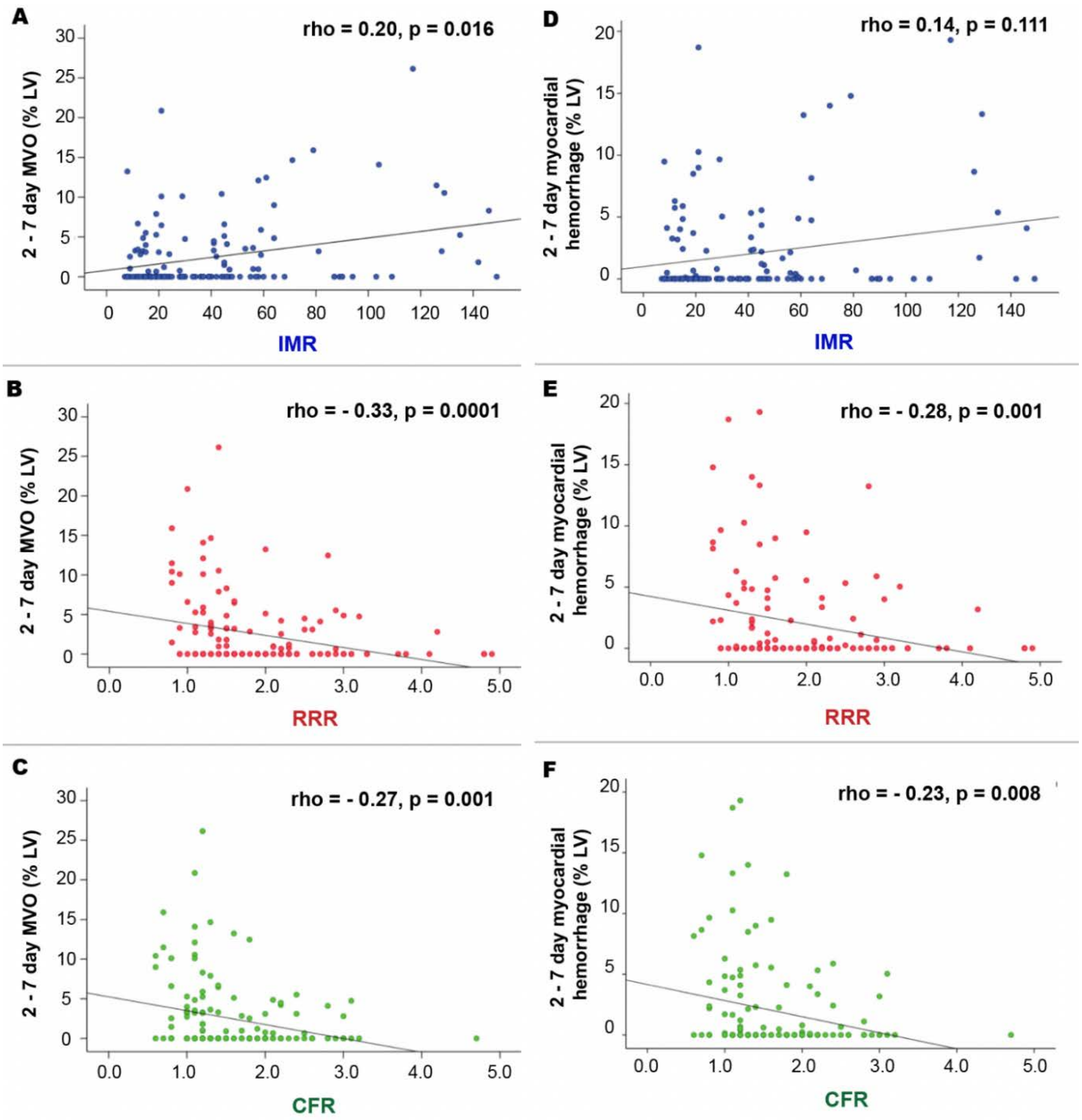

Scatterplots showing correlations between coronary physiology parameters (IMR [index of microcirculatory resistance]; RRR [resistive reserve ratio; CFR [coronary flow reserve]) with MVO (microvascular obstruction) (A, B and C) and with myocardial hemorrhage (D, E and F). LV, left ventricular.
